# Differences in energy metabolism and mitochondrial redox status account for the differences in propensity for developing obesity in rats fed on high‐fat diet

**DOI:** 10.1002/fsn3.2134

**Published:** 2021-01-23

**Authors:** Yipin Lu, Yingrui Li, Yongjuan Sun, Shuhua Ma, Kai Zhang, Xue Tang, Ailing Chen

**Affiliations:** ^1^ State Key Laboratory of Food Science and Technology Jiangnan University Wuxi China; ^2^ School of Food Science and Technology Jiangnan University Wuxi China; ^3^ National Engineering Research Center for Functional Food Jiangnan University Wuxi China; ^4^ Collaborative innovation center of food safety and quality control in Jiangsu Province Jiangnan University Wuxi China; ^5^ Translational Medicine Laboratory Research Institute for Reproductive Health and Genetic Diseases The Affiliated Wuxi Maternity and Child Health Care Hospital of Nanjing Medical University Wuxi China

## Abstract

Obesity is a metabolic disease that is accompanied by oxidative stress. Mitochondrial dysfunction is closely associated with the occurrence and development of obesity. However, it is unclear if there are differences in mitochondrial redox homeostasis and energy metabolism between obesity‐prone (OP) and obesity‐resistant (OR) individuals and if these differences account for the different susceptibilities to developing obesity. The present study aimed to compare the regulation of energy metabolism between OP and OR rats during high‐fat diet (HFD)‐induced oxidative stress. Male Sprague Dawley rats were randomly divided into the control group and the HFD group. The HFD group was further divided into the OP and OR groups based on body weight gain (upper 1/3 for OP; lower 1/3 for OR) after eight weeks on HFD. Rats were sacrificed at the 8th and 20th week, and serum and organs were collected. At 8 weeks, HFD decreased mitochondrial antioxidant enzyme activity and increased the production of ROS in the OP rats, which was accompanied by unusual mitochondrial oxidative phosphorylation, reduced mitochondrial membrane potential (MMP), and decreased ATP production. When the feeding period was extended beyond the 8 weeks, the energy expenditure of the OP rats reduced further, resulting in elevated blood lipids and glucose levels and increased body weight. In contrast, the OR rats had higher mitochondrial antioxidant enzyme activity and normal redox homeostasis throughout the period, which was beneficial in energy utilization and ATP production. Thus, the increase in energy expenditure in the OR rats reduced the HFD‐induced weight gain. Mitochondrial function and antioxidant defense might be involved in the different propensities for developing obesity. Consequently, the ability of OR rats to resist obesity may be attributed to their ability to maintain mitochondrial function and redox balance.

## Graphical abstract

Mitochondrial function and antioxidant defense might be involved in the different propensities for developing obesity. The ability of rats to resist obesity may be attributed to their ability to maintain mitochondrial function and redox balance.

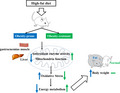

## 1 INTRODUCTION

Mitochondria are complex organelles that participate in many physiological processes, including ATP production and fatty acid oxidation (Vakifahmetoglu‐Norberg et al., 2017). Through oxidative phosphorylation (OXPHOS), mitochondrion generates not only ATP for cellular energy metabolism but also reactive oxygen species (ROS) (Nickel et al., 2014). Several studies have found that excessive intake of fat leads to excessive ROS production and induces oxidative stress(Milagro et al., 2006; Yu et al., 2019), which causes damage to mitochondria eventually leading to metabolic disorders (Balaban et al., 2005). Ji et al., 2015 found that ROS and MDA were significantly increased, total antioxidant capacity (T‐AOC) and superoxide dismutase (SOD) activity were significantly reduced, and mitochondrial lipid metabolism was abnormal in the liver of HFD‐induced obese rats. Adult rats on short‐term HFD showed obesity, insulin resistance, and hepatic steatosis, accompanied by increased mitochondrial oxidative stress and decreased OXPHOS in the liver (Raffaella et al., 2008). During the development of obesity, excessive ROS in skeletal muscle mitochondria induced insulin resistance (Barazzoni et al., 2012), which was associated with reducing mitochondrial oxidative capacity and ATP synthesis in skeletal muscles (Kelley et al., 1999). Therefore, maintaining mitochondrial redox homeostasis and function has great significance for the prevention and treatment of obesity and other metabolic diseases.

Obesity is characterized by a state of low‐grade chronic inflammation, and its incidence has increased worldwide. It is associated with type 2 diabetes mellitus, nonalcoholic fatty liver disease, cardiovascular disease, and various cancers (Bae et al., 2017). A particularly interesting aspect of obesity is the differences observed in response to HFD. One of the significant issues is the question of why some individuals are more susceptible to HFD‐induced obesity (obesity‐prone, OP) than others (obesity‐resistant, OR) (Wang et al., 2011). Studies on OP and OR mice found that OP mice were more likely to gain weight and body fat than OR mice, with a significant reduction in liver antioxidase and fatty acid oxidase enzymes (Oh, 2011). OP individuals are more likely to show elevated levels of insulin, glucose, and triglycerides, as well as reduced sensitivity to insulin and leptin, and decreased muscle metabolism (Astrup, 1993). OP and OR mice showed significant differences in several molecular pathways, including cAMP‐mediated signaling, hepatic fibrosis, and atherosclerosis signaling (Choi et al., 2016). Although the differences between OP and OR individuals have been studied in terms of proteomics, physiological signal, and neuromodulation (Kotz et al., 2008), there are few studies on the differences in mitochondrial redox homeostasis and energy metabolism. Therefore, this study aimed to induce obesity in rats using HFD and compare the periodical changes in the mitochondrial redox homeostasis and energy metabolism between the OP and OR rats. This will provide a theoretical basis for the prevention and treatment of obesity and its related diseases.

## 2 MATERIALS AND METHODS

### 2.1 animals

Sprague Dawley male rats (*n* = 84. 105.36 ± 0.62 g) that were 4‐week‐old were obtained from Shanghai SLAC Laboratory Animal Co., Ltd and housed in a controlled environment (a 12‐/12‐hr light/dark cycle, 08:00 hr to 20:00 hr, humidity: 40%–70%, temperature: 22–26°C). After acclimatization for one week on standard laboratory chow, all rats were randomly assigned into two groups and fed either a low‐fat diet (LFD, *n* = 21. 12.7% of calories from fat, energy density 3. 78 kcal/g) or a saturated high‐fat diet (HFD, *n* = 63, 45% of calories from fat, energy density 4.68 kcal/g). The detailed diet composition is shown in Table S1. All rats had free access to the test diets and purified water throughout the study. During the study period, all rats were weighed weekly, and food intake was also recorded. After eight weeks, rats that had been fed on the HFD were classified into OP and OR rats based on body weight. The rats (*n* = 21, 348.99 ± 17.00 g) in the upper tertile of body weight were numbered and selected as OP and those in the lower tertile (*n* = 21, 269.86 ± 13.61g) were grouped as OR. At 8, 14, and 20 weeks, 7 rats randomly selected from each of the Control, OP, and OR groups were sacrificed respectively after fasting for 12 hr to evaluate the effects of different periods of HFD feeding on the differentiation between OP and OR groups. The study was carried out according to the recommendations of the Guide for the Care and Use of Laboratory Animals of the Institutional Animal Care and Use Committee of Jiangnan University.

### 2.2 Indirect calorimetric analysis

The Comprehensive Laboratory Animal Monitoring System (C.L.A.M.S.; Columbus Instruments, Columbus, OH) was used to evaluate energy expenditure (EE), ambulatory activity, and food consumption. One week before the end of their respective dietary manipulations, the rats were placed in the C.L.A.M.S. for 24 hr for measurement of all in vivo parameters which included oxygen consumption, carbon dioxide production, and RER. The ambulatory activity was measured in both horizontal and vertical directions using infrared beams to count the beam breaks during the study. For each analysis, the animals were allowed to acclimatize in individual metabolic cages for one day and then the data of the second day were used for further analysis.

### 2.3 Blood and sample tissues preparation

After overnight fasting, the rats were anesthetized using ether, blood was taken from the heart, and tissues were dissected for further analysis. Fasting blood glucose (FBG) was assayed with a glucometer (One Touch; LifeScan, Milpitas, CA, USA). The liver, pancreas, soleus, and gastrocnemius tissues were carefully removed and individually weighed. Moreover, fat compartments, including retroperitoneal and epididymal fat, were carefully removed and weighed. The blood was centrifuged to obtain serum, which was then stored at −20°C until further analysis. All the tissues were frozen in liquid nitrogen and stored at −80°C until further analysis.

### 2.4 Isolation of mitochondria

Tissue samples (100 mg) were homogenized on ice immediately after harvest in 500 μl mitochondrial isolation buffer (Beyotime Institute of Biotechnology, Haimen, China) supplemented with phenylmethanesulfony fluoride (PMSF). The homogenate was centrifuged at 600 g, 4°C for 5 min. The supernatants were then collected gently and centrifuged at 11,000 g, 4°C for 10 min. The pellet mitochondria were collected, and the protein concentration was measured using a BCA protein assay kit (Institute of Biotechnology, Haimen, China).

### 2.5 Measurement of mitochondrial membrane potential (MMP)

The mitochondrial membrane potential in liver and gastrocnemius was measured using JC‐1 (Beyotime Biotechnology, Shanghai, China). A loss in mitochondrial membrane potential was shown by the increased green fluorescence from JC‐1 monomers as well as the reduction of red fluorescence from JC‐1 aggregates. The mitochondrial membrane potential was measured using a fluorescence spectrophotometer (FlourMax‐4, HORIBA, Tokyo, Japan).

### 2.6 Mitochondrial MnSOD and GSH‐Px activity assays

The activities of MnSOD were measured with WST‐8 assays after inhibiting the activity of Cu/ZnSOD using a specific inhibitor (Beyotime Biotech, Shanghai, China). Mitochondrial GPX activity was detected according to the manufacturer's instructions (Jiancheng Bioengineering Institute, Nanjing, China).

### 2.7 Acetyl coenzyme A, NADH, NAD+, and ATP content assay

The concentrations of acetyl coenzyme A, NADH/NAD+, and ATP in the tissues were measured using an ELISA kit (Huijia, Xiamen, China) and ATP determination kit (Beyotime Biotech, Shanghai, China), respectively. The concentration was normalized to that of protein in the same tissue lysates.

### 2.8 Measurement of serum parameters

Plasma total cholesterol (TC), triacylglycerol (TG), low‐density lipoprotein cholesterol (LDL‐C), and high‐density lipoprotein cholesterol (HDL‐C) levels were analyzed using enzymatic colorimetric assay kits (Nanjing Jiancheng Bioengineering Institute, Nanjing, China). Blood glucose and insulin were measured using a glucose determination kit (Jiancheng Bioengineering Institute, Nanjing, China) and an ELISA kit (Huijia, Xiamen, China), respectively. The insulin resistance index (HOMA‐IR) was calculated as HOMA‐IR = Insulin*glucose/22.5.

### 2.9 Determination of oxidative stress biomarkers in the blood and tissue samples

ROS was determined using a luminol‐dependent chemiluminescence assay in the presence of luminal (0.5 mmol/L) and horseradish peroxidase (12 U/mL) (Sigma) using a thermostatically (37°C) controlled luminometer (Xi'an Remex Analysis Instrument, Xi'an, China). ROS contents were expressed as relative light units (RLUs). The supernatants were tested using appropriate test kits (Jiancheng Bioengineering Institute, Nanjing, China). Oxidative stress biomarkers, including T‐AOC and MDA content in tissues and serum, were determined using kits obtained from Nanjing Jiancheng Bioengineering Institute (Nanjing, Jiangsu, China) according to the instructions of the manufacturer. The GSH and GSSG ratios were determined by Hissin & Hilf, 1976 using the OPT color reaction at a fluorescence excitation wavelength of 350 nm and an emission wavelength of 430 nm.

### 2.10 Statistical analysis

Data are presented as mean ± *SEM*. Differences between two groups were analyzed by one‐way analysis of variance test followed by Tukey's test.

## 3 RESULTS AND DISCUSSION

### 3.1 High‐fat diet‐induced OP and OR phenotypes

**FIGURE 1:**
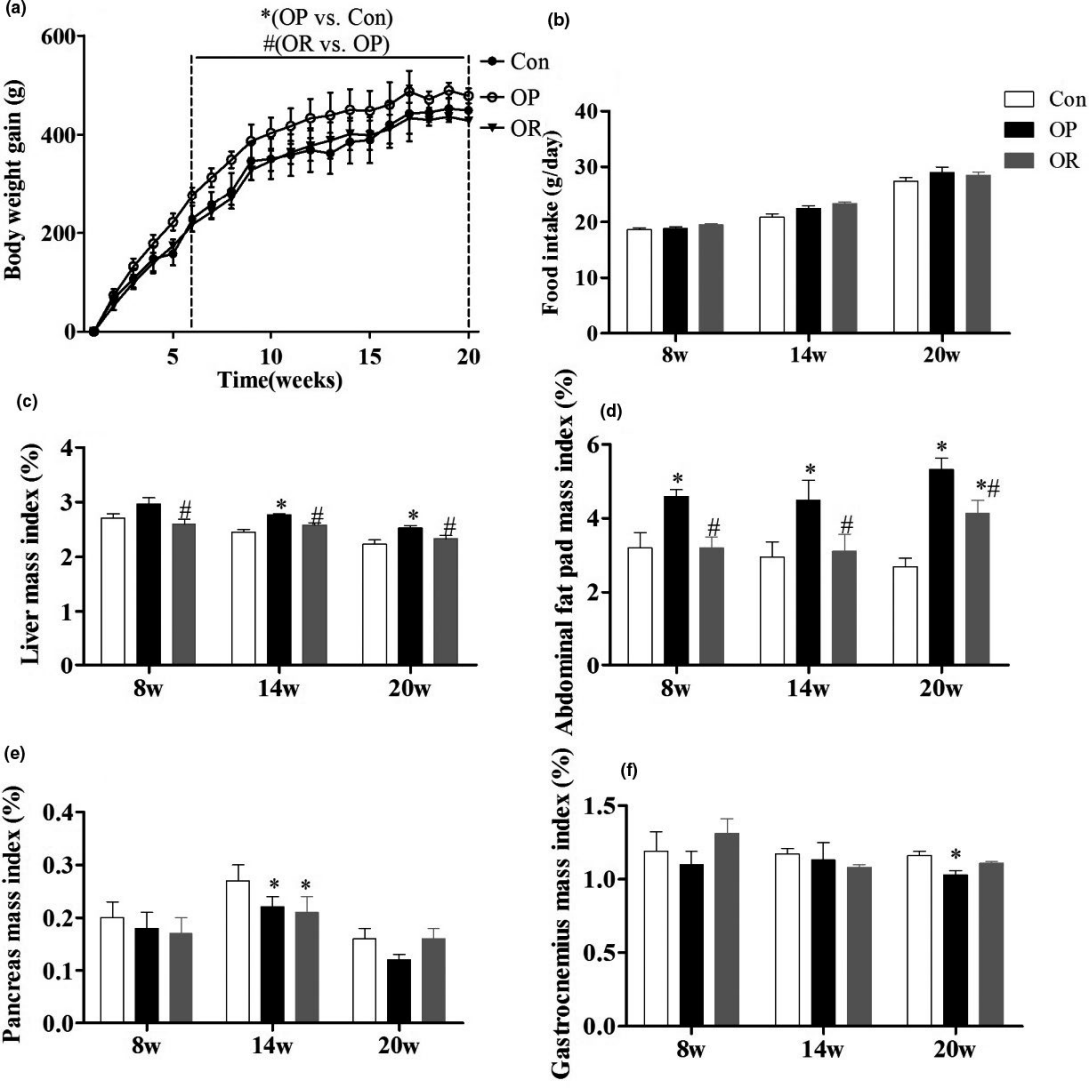
Phenotypes of OP and OR rats induced by HFD. (a) Body weight gain. (b) Food intake. (c) Liver mass index (g/g body weight). (d) Abdominal fat pad mass index. (e) Pancreas mass index. (f) Gastrocnemius mass index. Data were presented as mean ± *SEM*. Con, control group; OP, obesity‐prone; OR, obesity‐resistant. *p < .05 versus Con;^#^ *p* < .05 versus OP

From the beginning of the study until the 6th week, there was no significant difference in the gain in body weight among the three groups of rats. After the 6th week, the rate of gain in body weight among the OP rats was significantly faster than that of the controls and OR rats. This significant difference in body weight gain (Figure 1a) continued to increase during the subsequent few weeks (*p* < .05), while there was no significant difference between the OR group and the Control group (*p* > .05). However, there was no significant difference in feed intake (Figure 1b) among the three groups (*p* > .05). Figure 1c‐d shows that the liver mass index (at 14 weeks and 20 weeks) and abdominal fat pad mass index (at 8 weeks, 14 weeks and 20 weeks) in the OP group were significantly higher than those in the Control and OR groups (*p* < .05). In addition, there was no significant difference in abdominal fat pad mass index between the OR and Control rats except at 20 weeks. These results on abdominal fat pad mass index indicate that the HFD‐induced gain in body weight was mainly caused by fat accumulation. There was no significant change in the pancreas and gastrocnemius mass index (Figure 1e‐f) among the three groups. There was a significant decrease in the pancreatic mass index in the OP and OR groups as well as the gastrocnemius mass index in the OP group at 14 and 20 weeks, respectively (*p* < .05).

### 3.2 Differences in energy intake, energy expenditure, oxygen consumption, and activity between OP and OR rats

**FIGURE 2:**
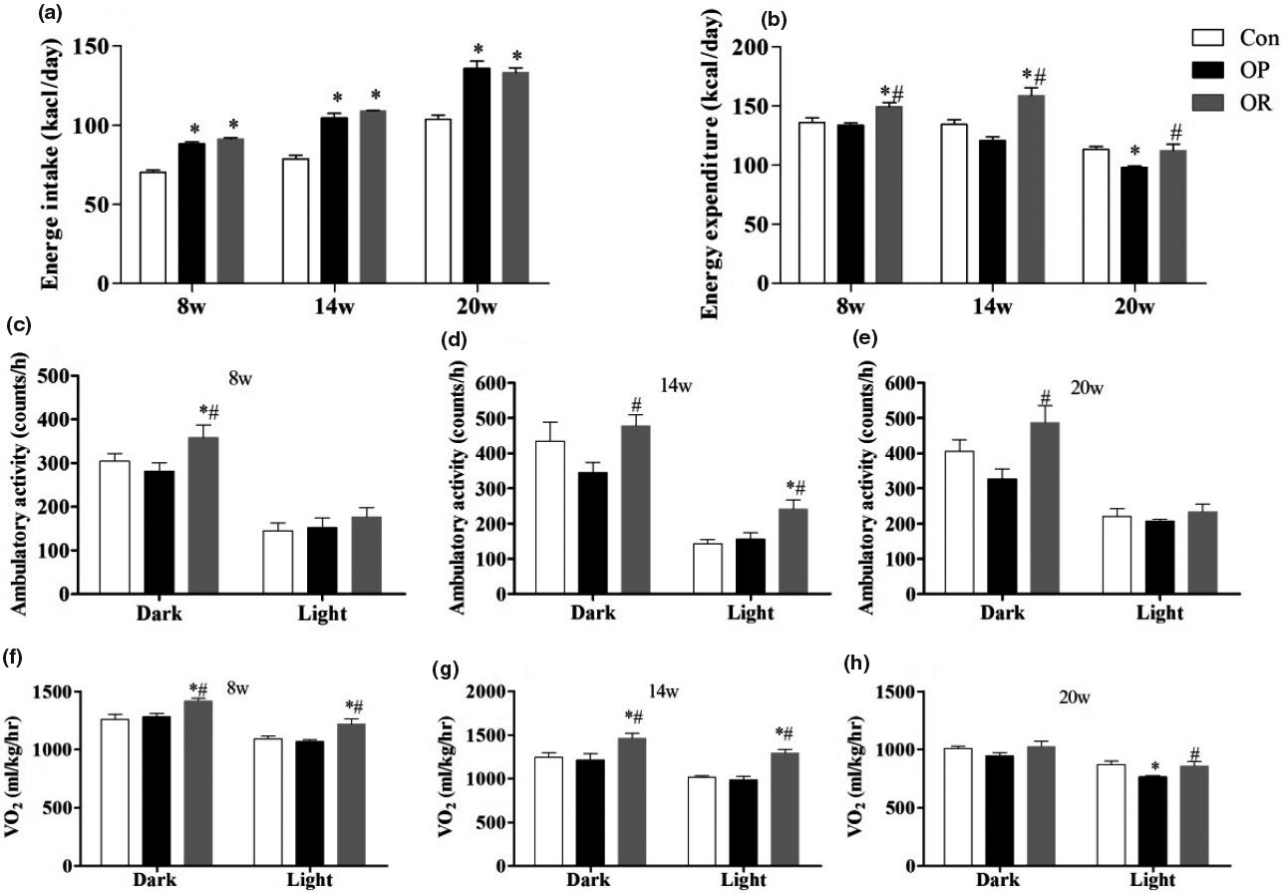
Indirect calorimetry in Con, OP and OR rats in the 8th week, 14th, and 20th week. (a) Energy intake, (b) Energy expenditure, (c‐e) Ambulatory activity, (f‐h) Oxygen consumption. Bar graphs represent mean values during the light and dark cycles. Data were presented as mean ± *SEM*. Con, control group; OP, obesity‐prone; OR, obesity‐resistant. *p < .05 versus Con; ^#w^ *p* < .05 versus OP

As shown in Figure 2a, the energy intake in the OP and the OR groups was significantly higher than in the Control group during the entire 20 weeks (*p* < .05), but there was no significant difference between the OP group and the OR group. There was also no significant difference in the energy expenditure between the Control and OP group from the 8th week to the 14th week (*p* > .05), but at 20 weeks, there was a significant decrease in energy expenditure in the OP group (*p* < .05) (Figure 2b). On the other hand, the energy expenditure in the OR group at 8 and 14 weeks was significantly higher than that of the Control and the OP group (*p* < .05). At 20 weeks, although the energy consumption of the OR group was still significantly higher than that of the OP group (*p* < .05), there was no significant difference with the Control group (*p* > .05). In addition, the OR group had significantly higher ambulatory activity (Figure 2c‐e) and oxygen consumption (Figure 2f‐h) compared to the OP group throughout the entire feeding period (*p* < .05). This indicates that ambulatory activity and oxygen consumption might play a role in the energy expenditure of OR rats.

### 3.3 Differences in the activity of mitochondrial antioxidant enzymes in the liver, gastrocnemius, and pancreas between OP and OR rats

**FIGURE 3:**
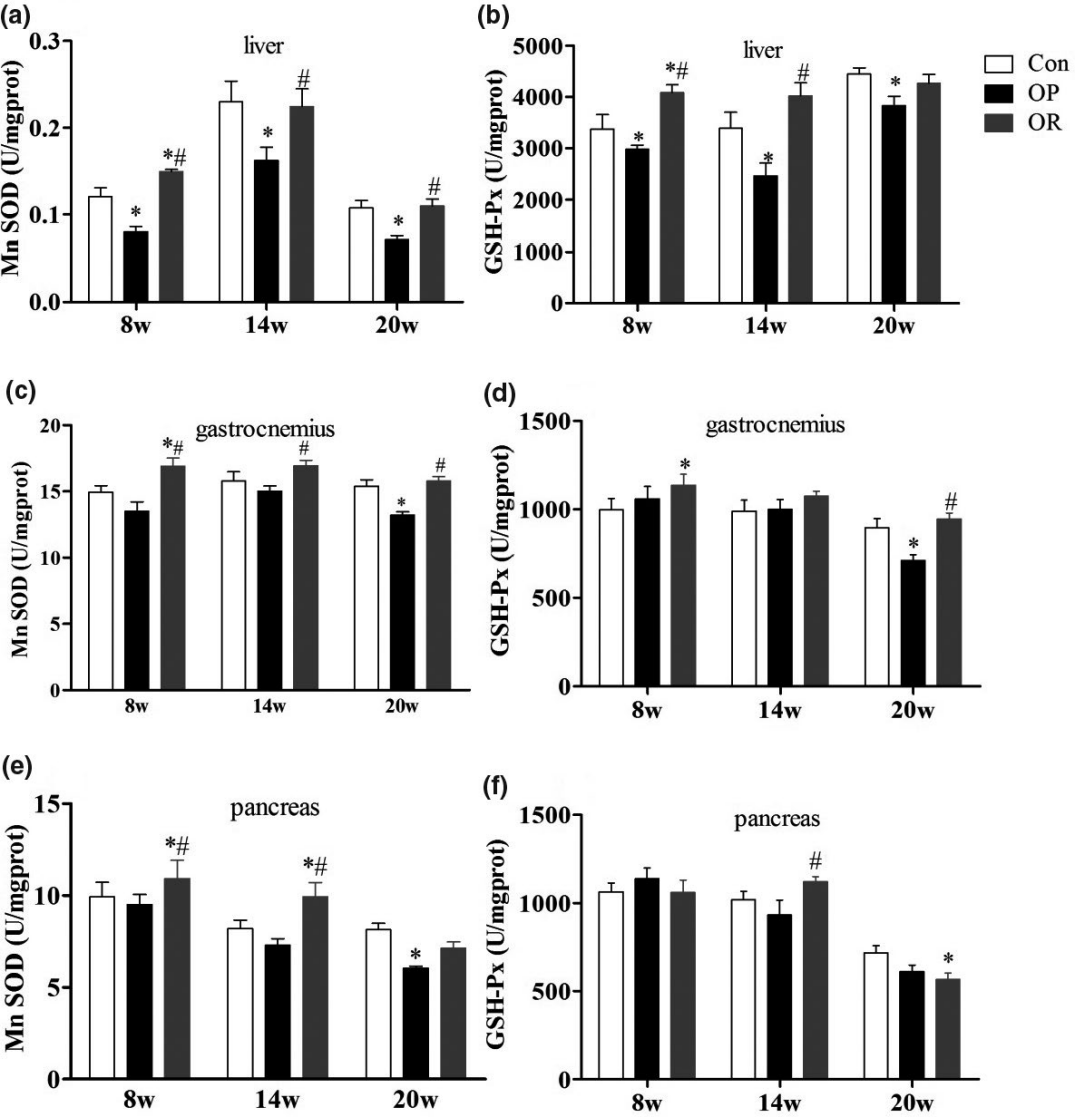
Differences of mitochondrial antioxidant enzymes among Con, OP, and OR rats. (a‐b) liver, (c‐d) gastrocnemius, (e‐f) pancreas. Data were presented as mean ± *SEM*. Con, control group; OP, obesity‐prone; OR, obesity‐resistant. *p < .05 versus Con; ^#^ *p* < .05 versus OP

We analyzed the changes in the activities of mitochondrial antioxidant enzymes in the essential organs of glucose and lipid metabolism, such as the liver, pancreas, and gastrocnemius, at different time points. As shown in Figure 3, at 8 weeks, the activities of MnSOD and GSH‐Px in the mitochondria of the liver and gastrocnemius muscle were significantly increased in the OR group compared to the Control group (*p* < .05). Meanwhile, MnSOD activity in the pancreas increased significantly in the OR group compared to the Control group (*p* < .05). On the contrary, the activity of mitochondrial enzymes in the liver of the OP rats was significantly lower than that of the Control rats (*p* < .05), but there was no significant difference in the enzyme activity in the other two tissues (*p* > .05). At 20 weeks, the activities of mitochondrial antioxidant enzymes were still higher in the OR rats compared to the control group, while there was a significant decrease in the activity of the enzymes in all three tissues in OP rats, which was particularly significant in the liver and gastrocnemius (*p* < .05).

### 3.4 Differences in the levels of oxidative stress biomarkers in liver, gastrocnemius, and serum between OP and OR rats

As shown in Table 1, the levels of T‐AOC in the liver and GSH/GSSG in the gastrocnemius of OP rats were significantly lower than those of the Control rats, while the levels of ROS and MDA were significantly higher at 8 weeks (*p* < .05). At the same time, the four oxidative stress parameters showed significant differences in the serum of OP rats compared to the Control group (*p* < .05), indicating that the OP rats had begun to suffer from oxidative stress. Surprisingly, the OR rats had significantly elevated levels of T‐AOC and GSH/GSSG compared to the OP rats (*p* < .05), indicating that the OR rats had enhanced antioxidant capacity in the early stages of the study. When the feeding period was extended to 20 weeks, the OP rats had severe oxidative stress as indicated by the significantly low levels of T‐AOC and GSH/GSSG and higher accumulation of ROS and MDA levels in serum, liver, and gastrocnemius compared to the Control rats (*p* < .05). On the other hand, the oxidative stress parameters were normal in the serum of the OR rats but slightly abnormal in the liver and gastrocnemius, suggesting that OR rats could more efficiently defend against oxidative stress.

**TABLE 1:** Oxidative stress biomarkers in liver, gastrocnemius, and serum of rats fed the standard chow or HFD for 8, 14, and 20 weeks

Diet type	Week8			Week14			Week20		
	Con	OP	OR	Con	OP	OR	Con	OP	OR
**Liver**									
T‐AOC (U/mg prot)	0.88 ± 0.06	0.76 ± 0.12^*^	0.81 ± 0.06	0.90 ± 0.15	0.75 ± 0.14^*^	1.01 ± 0.18^#^	0.71 ± 0.10	0.55 ± 0.07	0.67 ± 0.10^#^
MDA (U/mg prot)	2.14 ± 0.51	2.76 ± 0.20^*^	2.43 ± 0.53^#^	2.02 ± 0.11	3.37 ± 0.33^*^	2.25 ± 0.37^#^	2.02 ± 0.39	4.25 ± 0.39^*^	2.27 ± 0.48^#^
GSH/GSSG	3.19 ± 0.28	2.99 ± 0.22	3.54 ± 0.35	2.38 ± 0.24	2.18 ± 0.12	3.31 ± 0.18^*#^	2.62 ± 0.32	1.91 ± 0.07^*^	2.27 ± 0.19
ROS (10^3^AUC/μl)	1.01 ± 0.06	1.52 ± 0.63^*^	0.77 ± 0.18^*#^	1.03 ± 0.06	1.53 ± 0.38^*^	1.30 ± 0.25	1.02 ± 0.21	1.89 ± 0.74^*^	1.74 ± 0.95^*^
**Gastrocnemius**									
T‐AOC (U/mg prot)	0.27 ± 0.04	0.25 ± 0.03	0.38 ± 0.06^*#^	0.37 ± 0.08	0.28 ± 0.07^*^	0.47 ± 0.03^#^	1.10 ± 0.08	0.76 ± 0.03^*^	1.02 ± 0.05
MDA (U/mg prot)	1.78 ± 0.22	2.58 ± 0.31^*^	1.79 ± 0.44^#^	3.94 ± 0.64	4.83 ± 0.69^*^	3.56 ± 0.82^#^	2.72 ± 0.51	3.93 ± 0.51^*^	3.22 ± 0.13^#^
GSH/GSSG	4.66 ± 0.4	4.02 ± 0.24^*^	4.45 ± 0.21^#^	4.61 ± 0.29	4.65 ± 0.25	4.76 ± 0.34	6.45 ± 0.24	5.74 ± 0.12^*^	6.02 ± 0.06
ROS (10^3^AUC/ul)	1.03 ± 0.13	1.13 ± 0.18	0.90 ± 0.11^#^	1.01 ± 0.29	1.27 ± 0.19^*^	1.09 ± 0.25	1.01 ± 0.14	1.35 ± 0.08^*^	1.28 ± 0.07^*^
**Serum**									
T‐AOC (U/mg prot)	5.33 ± 0.34	4.49 ± 0.23^*^	4.79 ± 0.24	5.60 ± 0.21	5.04 ± 0.35^*^	5.68 ± 0.14^#^	5.44 ± 0.19	4.14 ± 0.20^*^	5.18 ± 0.37^#^
MDA (U/mg prot)	4.90 ± 0.41	6.12 ± 0.38^*^	5.44 ± 0.57	4.90 ± 0.51	6.35 ± 0.56^*^	6.25 ± 0.29^*^	2.56 ± 0.54	4.17 ± 0.35^*^	3.15 ± 0.42^#^
GSH/GSSG	7.58 ± 0.14	4.07 ± 0.16^*^	6.49 ± 0.20^#^	7.56 ± 0.34	5.46 ± 0.24^*^	7.47 ± 0.31^#^	7.12 ± 0.29	5.43 ± 0.14^*^	6.89 ± 0.11^#^
ROS (10^3^AUC/μl)	1.02 ± 0.14	1.27 ± 0.22^*^	1.06 ± 0.25	1.10 ± 0.16	1.30 ± 0.24^*^	1.25 ± 0.32	1.00 ± 0.28	1.45 ± 0.37^*^	1.16 ± 0.35^#^

Note

Data were presented as mean ± *SEM*. Con, control group; OP, obesity‐prone; OR, obesity‐resistant. ^*^ *p* < .05 versus Con; ^#^ *p* < .05 versus OP.

### 3.5 Differences in mitochondrial energy metabolism in liver and gastrocnemius between OP and OR rats

**FIGURE 4:**
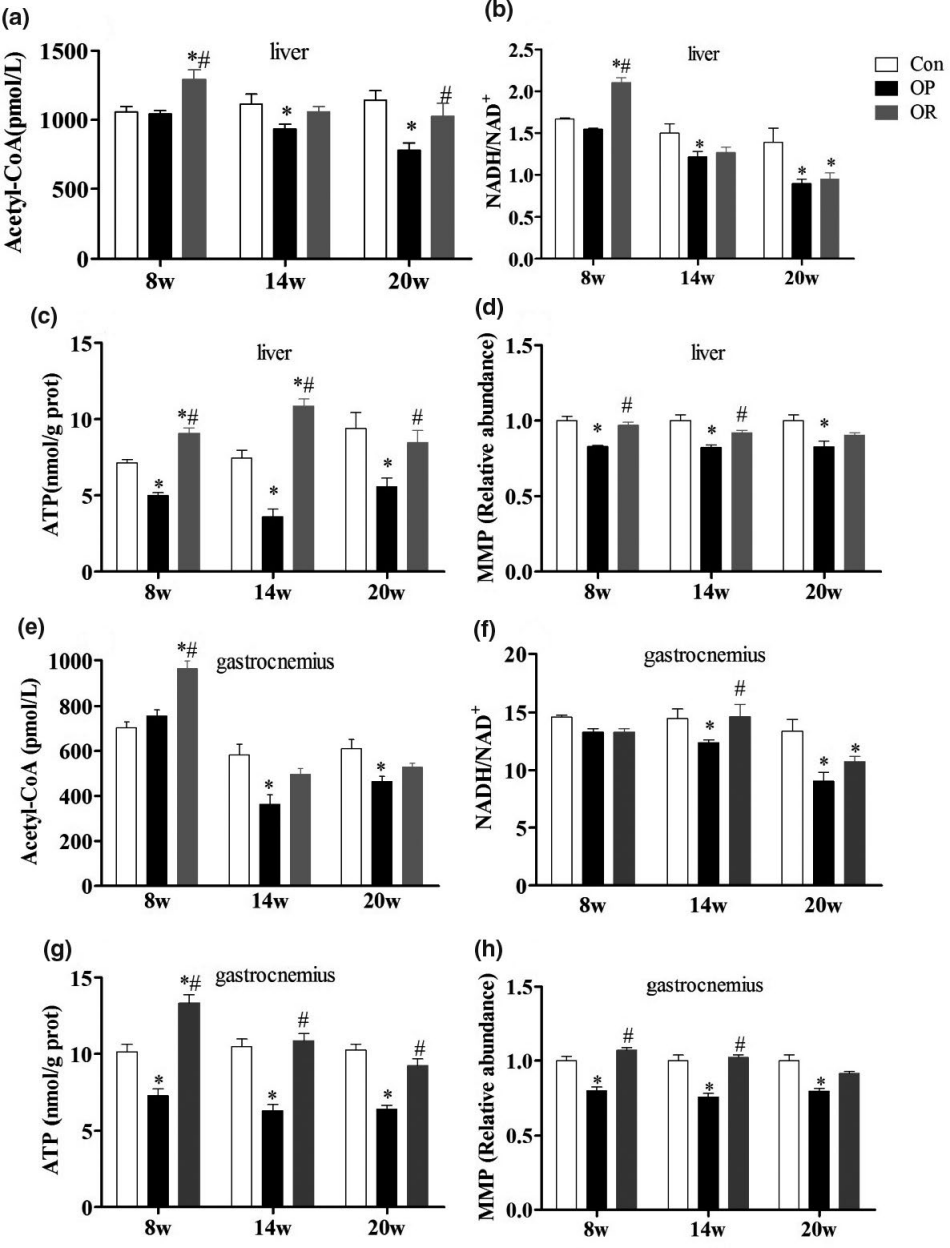
Differences of mitochondrial energy metabolism in liver and gastrocnemius among Con, OP, and OR rats. (a‐d) liver, (e‐h) gastrocnemius. Data were presented as mean ± *SEM*. Con, control group; OP, obesity‐prone; OR, obesity‐resistant. *p < .05 versus Con; ^#^ *p* < .05 versus OP

As shown in Figure 4, there was a significant decrease in the hepatic mitochondrial acetyl CoA content and NADH/NAD + ratio in the OP rats compared to the control rats (*p* < .05) after HFD for 14–20 weeks, further leading to the decrease of MMP and ATP production (*p* < .05). However, there was a significant increase in the hepatic mitochondrial ATP production of OP rats compared to the control mice (*p* < .05) from the 8th week to 14th week but there was no statistical difference from the 14th week to the 20th week. In addition, there was a significant difference in the mitochondrial ATP production and MMP of gastrocnemius in the OP group compared to the control group at 14 and 20 weeks.

### 3.6 Differences in serum parameters between OP and OR rats

As shown in Table 2, there was a significant increase in the blood lipid indicators (TG and TC) in the OP group compared to the Control group at 8 weeks, indicating that HFD was successful in inducing obesity in the rat model The levels of TG ( at the 8th, 14th, and 20th week), TC, and LDL‐C(at the 20th week) were significantly higher in the OP group compared to the Control group (*p* < .05), while there were no differences observed between the OR rats and the Control rats (*p* > .05). The results showed that OP rats developed dyslipidemia at the beginning of HFD feeding and further deteriorated with the extension of the cycle. In contrast, OR rats maintained normal levels throughout the study. At 8 weeks of HFD, the levels of insulin, blood glucose, and HOMA‐IR were significantly reduced in the OP rats (*p* < .05), but there was no significant difference in the levels compared to the OR rats (*p* > .05). After 14 to 20 weeks of HFD, the levels of insulin, blood glucose, and HOMA‐IR were significantly higher in the OP rats compared to the control rats (*p* < .05), while there was no significant difference with the OR rats (*p* > .05). The results of this study showed that the OP rats were able to maintain normal blood glucose levels during short‐term (8 weeks) feeding with HFD, but the regulation of blood glucose was disordered as the feeding period was prolonged. On the contrary, the OR rats showed better ability to maintain blood glucose levels throughout the study period.

**TABLE 2:** Clinical and biochemical parameters of rats fed the standard chow or HFD

	TG (mmol/L)	TC (mmol/L)	HDL‐C (mmol/L)	LDL‐C (mmol/L)	Insulin (mU/L)	Glucose (mmol/L)	HOMA‐IR
Week 8							
Con	0.95 ± 0.21	1.23 ± 0.11	0.35 ± 0.08	0.24 ± 0.04	9.75 ± 0.68	9.34 ± 0.65	4.05 ± 0.11
OP	1.71 ± 0.11^*^	1.66 ± 0.16^*^	0.33 ± 0.05	0.32 ± 0.10	8.38 ± 0.16^*^	7.85 ± 0.30^*^	2.92 ± 0.28^*^
OR	1.32 ± 0.13	1.40 ± 0.08	0.37 ± 0.04	0.27 ± 0.64	9.03 ± 0.98	8.03 ± 0.29	3.18 ± 0.13^*^
Week 14							
Con	0.75 ± 0.06	1.14 ± 0.10	0.36 ± 0.03	0.38 ± 0.01	12.85 ± 0.87	7.91 ± 0.76	4.52 ± 0.43
OP	1.04 ± 0.09^*^	1.39 ± 0.11	0.31 ± 0.03	0.38 ± 0.03	12.54 ± 0.37	10.79 ± 0.88^*^	6.01 ± 0.49^*^
OR	0.79 ± 0.04^#^	1.25 ± 0.08	0.38 ± 0.04	0.37 ± 0.02	10.65 ± 0.48^*#^	9.67 ± 0.56^*^	4.58 ± 0.27^#^
Week 20							
Con	0.87 ± 0.03	1.25 ± 0.11	0.53 ± 0.06	0.14 ± 0.01	12.22 ± 0.35	7.99 ± 0.32 ^a^	4.34 ± 0.17
OP	1.13 ± 0.06^*^	1.52 ± 0.07^*^	0.52 ± 0.03	0.18 ± 0.01^*^	14.57 ± 0.51^*^	9.67 ± 0.26 ^*^	5.61 ± 0.17^*^
OR	0.86 ± 0.08^#^	1.26 ± 0.05^#^	0.56 ± 0.05	0.15 ± 0.01^#^	11.80 ± 0.79^#^	8.62 ± 0.08	4.52 ± 0.04^#^

Note

Data were presented as mean ± *SEM*. Con, control group; OP, obesity‐prone; OR, obesity‐resistant. *p < .05 versus Con; ^#^ *p* < .05 versus OP

## 4 DISCUSSION

The main findings of the present study were that the different propensities for developing obesity after short‐term and long‐term HFD feeding might be associated with oxidative stress induced by mitochondrial dysfunction. In the early stages of HFD, OR rats enhanced mitochondrial energy expenditure due to normal redox homeostasis maintained by improving mitochondrial antioxidant enzyme activity (MnSOD and GSH‐Px). This allowed them to avoid hyperlipidemia, hyperglycemia, and rapid weight gain.

Energy intake that is greater than energy expenditure is the leading cause of obesity (Schmidt et al., 2013). For the same amount of food given, the higher the fat content, the higher the energy density. Therefore, feeding animals with HFD to induce obesity is an ideal model for exploring the mechanism of human obesity (Lin et al., 2000; Woods et al., 2003). Studies have shown that HFD can induce the accumulation of body fat, but different individuals have different susceptibility to HFD‐induced obesity (Huang et al., 2004). The body weight curve obtained from this study suggests that the OP rats are more likely to gain weight than the OR rats, which is consistent with the findings of Choi et al., 2010. The present study showed that OR rats maintained low body weight gain, liver mass index, and abdominal fat mass index to delay the occurrence and development of obesity compared with the OP rats. Howard et al., 1991 pointed out that OP individuals are more likely to gain weight due to low energy consumption, which is in contrast to the OR individuals who use more fat and consume more energy. Therefore, we used the CLAMS system to monitor the metabolism of the rats in each group. We found that HFD significantly increased the energy intake of the OP and OR groups compared to the Control group. Although there was no significant difference in energy intake between the OP and OR groups, the energy expenditure in the OR group was significantly higher than that of the OP group. At the same time, we also observed that the ambulatory activity and oxygen consumption were significantly higher in the OR group compared to the OP group, and that the OR rats showed better metabolic flexibility. Teske et al., 2012 reported that an increase in the ambulatory activity of OR rats led to decreased body weight and fat accumulation, revealing that increased energy expenditure resulting from increased ambulatory activity is one of the factors that contributes to the differences in susceptibility to obesity. About 90% of oxygen consumption occurs in the mitochondria, 80% of which is associated with ATP synthesis (Rolfe & Brown, 1997). The body can enhance mitochondrial respiration and increase energy utilization through increased oxygen consumption (Hong et al., 2015). Mitochondria are the sites of energy metabolism and respiration. Therefore, we analyzed the mitochondrial function of the main organs involved in glucose and lipid metabolism.

Mitochondrial function and its antioxidant capacity are closely related (Zou et al., 2016). MnSOD and GSH‐Px are crucial antioxidant enzymes in the mitochondria(Tang et al., 2020). When electrons are transported through the electron transport chain via NADH, mitochondrial complexes I and II produce O2∙ in the mitochondrial matrix, while complex III produces O2 ∙ in the matrix and intima (Murphy, 2009). O2∙ is a type of ROS that is highly reactive and can destroy and inactivate iron–sulfur cluster‐containing proteins (Outten, 2007). O2∙ is broken down into hydrogen peroxide (H_2_O_2_) by MnSOD. However, the accumulation of H_2_O_2_ can also be detrimental, as it results in aberrant signaling or is reduced to harmful hydroxyl radicals (OH∙). To prevent these detrimental effects, H_2_O_2_ is broken down into H_2_O and O_2_ by GSH‐Px and in the process GSH is oxidized to GSSG. (Jones et al., 1995). In this case, GSH acts as an intracellular non‐enzymatic antioxidant. T‐AOC and GSH/GSSG are essential indicators of redox status. MDA is a product of lipid oxidation when the body is under oxidative stress and is widely used to measure lipid peroxidation. In the early stages of HFD feeding, the OP rats began to suffer from oxidative stress, while the OR rats enhanced their antioxidant capacity. When the feeding period was extended to 20 weeks, the OP rats had severe oxidative stress while the OR rats were efficiently able to defend against oxidative stress. Xia et al., 2015 found that after seven weeks and 27 weeks HFD feeding, hepatic GSH‐Px and SOD activity increased significantly and MDA decreased significantly in OR mice compared to OP mice, indicating that OR mice had more potent antioxidant capacity. This is consistent with the results from our study. Thu et al., 2010 found that the knockout of MnSOD or GSH‐Px in mice increased the production of mitochondrial ROS in cardiomyocytes, resulting in oxidative damage and mitochondrial dysfunction. On the other hand, the overexpression of MnSOD and GSH‐Px effectively enhanced mitochondrial antioxidant capacity and restored oxidative stress‐induced mitochondrial dysfunction (Jang et al., 2009). Our results suggest that during short‐term (8 weeks) feeding with HFD, the OR rats enhance mitochondrial antioxidant capacity and further improve redox homeostasis by increasing the activities of MnSOD and GSH‐Px. Some phytochemicals in food are natural antioxidants (Chen, Tan, et al., 2018), such as tea polyphenols (polyphenols), soybean isoflavones (flavonoids), lycopene (carotenoids), and vitamins (Chen et al., 2019). Both in vivo and in vitro experiments confirmed that these substances can reduce the level of ROS (Chen, Zhou, et al., 2018; Silvia et al., 2008) and may affect the occurrence and development of obesity.

Redox homeostasis has a crucial impact on mitochondrial function and whole‐body metabolism. The liver and gastrocnemius are the main sites of glucose and lipid metabolism, and ATP, acetyl CoA, and NADH/NAD + ratio are vital markers of mitochondrial energy metabolism (Aizawa et al., 2016). Besides, the mitochondrial membrane potential (MMP) formed by mitochondrial oxidative phosphorylation is necessary for maintaining the exchange of substances inside and outside the mitochondria and is used to evaluate mitochondrial function (Zanella et al., 2011). Xing et al., 2016 observed a decrease in the ratio of NADH/NAD + and acetyl CoA in HepG2 cells induced by H_2_O_2_‐induced oxidative damage, suggesting that mitochondrial energy metabolism was inhibited. However, decreased MMP and ATP production indicates that the mitochondrial normal oxidative phosphorylation pathway was inhibited (Tasaka et al., 2008). Our results suggest that short‐term HFD caused abnormalities in the oxidative phosphorylation of the mitochondrial respiratory chain, and decreased MMP and ATP production in the OP rats, which further led to disorders in mitochondrial energy metabolism. Therefore, OP rats developed dyslipidemia at the beginning of HFD feeding and further deteriorated with the extension of the period. In the short‐term (8 weeks) with HFD feeding, OP rats were able to maintain normal blood glucose levels, but prolonged feeding disordered the blood glucose levels. However, the OR rats maintained normal blood lipids and blood glucose levels throughout the study period due to their ability to enhance mitochondrial energy metabolism.

## 5 CONCLUSION

In conclusion, the different propensities for developing obesity may be associated with the differences in energy metabolism and mitochondrial antioxidant enzyme activity. Increased mitochondrial antioxidant enzyme activity during the early stages of HFD feeding enabled the OP rats to maintain redox homeostasis and thus facilitated mitochondrial energy metabolism to maintain energy balance. However, as the HFD feeding period was prolonged, this antioxidant feedback regulation mechanism in OR rats diminished gradually, leading to the onset of oxidative stress. In the late stage of HFD feeding, OP rats already had severe oxidative stress, impaired mitochondrial function, and disturbed glucose and lipid metabolism, with a continuous increase in body weight. Therefore, in the early stage, the redox status was regulated by enhancing mitochondrial antioxidant capacity, which was of significance for maintaining the body's energy metabolism and slowing down the occurrence and development of obesity.

## CONFLICTS OF INTEREST

The authors have declared that no conflict of interest exists.

## Supporting information

Table S1
