# Supplementary material for: Differences in energy metabolism and mitochondrial redox status account for the differences in propensity for developing obesity in rats fed on high‐fat diet
Source: Food Sci Nutr. 2021 Jan 23;9(3):1603–13. doi: 10.1002/fsn3.2134 (PMC7958544; doi:10.1002/fsn3.2134)
Supplement: Supplementary file 1 — Table S1 [file FSN3-9-1603-s001.docx]

**Table S1.** Compositions of normal and high fat diet

| Component | NFD% | HFD% | Component | NFD% | HFD% |
| --- | --- | --- | --- | --- | --- |
| Corn Flour | 49.92 | 30.30 | Methionine | 0.20 | 0.20 |
| Wheat Flour | 9.00 | 9.00 | CaHPO_4_ | 1.20 | 1.20 |
| Sucrose | 0.10 | 0.10 | CaCO_3_ | 1.60 | 1.60 |
| Wheat Bran | 9.00 | 9.00 | NaCl | 0.20 | 0.20 |
| Soybean Oil | 2.80 | 2.80 | Vitamin | 0.02 | 0.02 |
| Lard | 1.00 | 20.62 | Choline Chloride | 0.10 | 0.10 |
| Soybean Meal | 24.52 | 24.52 | Minerals | 0.06 | 0.06 |
| Lysine | 0.28 | 0.28 |  |  |  |
